# Does cultural resource endowment backfire? Evidence from China’s cultural resource curse

**DOI:** 10.3389/fpsyg.2023.1110379

**Published:** 2023-01-30

**Authors:** Jianxin Zhou, Zhen Xia, Yongshi Lao

**Affiliations:** ^1^Institute for Cultural Industries, Shenzhen University, Shenzhen, China; ^2^Shenzhen Qijing Consulting Services Limited Company, Shenzhen, China; ^3^School of Management, Shenzhen University, Shenzhen, China

**Keywords:** resource curse theory, cultural resource endowment, cultural resource curse, cultural industries, transmission mechanism

## Abstract

Resource curse theory suggests that regions rich in natural resource endowments accumulate adverse economic competitive, but few studies have focused on causes and mechanisms of cultural resource curses. Since the development of the cultural industries is relatively backward in some regions with rich cultural resources in central and western China. Combined with the theory of cultural resources and the resource curse, we build cultural resource endowment and cultural resource curse coefficients and measure the distribution of cultural resource curses based on the dataset of 29 provinces in China covering 2000−2019. The results show that there is a serious cultural resource curse in western China. The causes of the cultural resource curse are multiple, place attachment and cultural field can influence cultural behaviors, and the environmental impact of industrial ecosystems causes path dependence in cultural resource exploration and cultural industry development. We further empirically tested the influence of cultural resources on cultural industries in different sub-regions of China and the transmission mechanism of the curse of cultural resources in western China. The results show that the influence of cultural resources on the cultural industries is not significant in the overall of China, but it is significantly negative in western China. The resource-dependent model of cultural industries development in western China has attracted more primary labor and crowded out government spending on education. Moreover, it hinders the upgrading of human resources and inhibits the modern innovative development of the cultural industries. This is an important reason for the curse of cultural resources in the development of cultural industries in western China.

## 1. Introduction

In the field of cultural industries, cultural resources can be applied to economic transactions, such as cultural destination development (Bourdieu, 1983). A long time in human civilization constitutes, many traditional material cultural resources and intangible cultural resources that have existed for an important endowment of human civilization for the development of cultural industries (King, 2011; Li and Katsumata, 2020). However, empirical facts show that the development of cultural industries in areas enriched with cultural resources lags far behind that in areas where cultural resources are poor. For example, western China, which has been on the Silk Road (SR) since ancient times, has a long historical and cultural tradition and rich cultural resources, but its cultural industry development lags behind some areas in eastern China. On the other hand, Shenzhen in eastern China, once likened to a cultural desert, was a small fishing village 40 years ago, and now the cultural industries are developing strongly. Scholars have established an important driving force for the modernization of cultural industries elements such as social capital (Chuluunbaatar et al., 2014), artificial intelligence and big data (Qin and Lin, 2021), digital serialization (McMullen et al., 2021), creative workers (Shaughnessy et al., 2022). Due to the heterogeneity of the composition of cultural resources, the transformation of the development mode of modern cultural industries is based on contemporary factors. We consider whether the traditional endowments of these historical and cultural resources have become a burden on the development of the cultural industries, and how they further affect the resource allocation behavior in the development of the cultural industries. In response to this problem, the current study has drawn on the resource curse theory to examine the cultural resource curse in the development of cultural industries.

Taking into account the differences in economic development levels and resource endowments in different regions, the resource curse theory explains the poor growth experience of resource-rich countries through natural resources to drive development (Sachs and Warner, 2001; Auty, 2007; Wen and Jia, 2022), extant studies have attempted to elucidate the causes and mechanisms of this curse such as the decline in the primary commodity terms of trade (Ross, 1999), differences in the quality of institutions (Mehlum et al., 2006), political corruption and the quality of politicians (Brollo et al., 2013), property rights (Wenar, 2012), links between natural resource rents and financial development (Li Z. et al., 2021). However, previous research on the resource curse mainly focuses on natural resources and economic growth, while the research on the cultural resource curse has received less scholarly attention. In this study. we aim to explore the causes of the transmission mechanism, and the curse of cultural resources. To test our hypotheses, we collected data from 29 provinces in China covering 2000−2019.

Drawing on the theory of the resource curse, this study aims to empirically explain the deviation between cultural industries and cultural resource endowment, and specifically examines the cultural resource curse in the emerging economy (such as China). More specifically, we first developed the cultural resource endowment index, which defined the scope of cultural resource endowment, including public cultural resources, cultural heritage resources, tourism resources, local language resources, and famous villages and towns resources. Second, based on the regional cultural resource endowment and the comparative advantage of cultural industries development, the cultural resource curse coefficient is calculated to explore the regional distribution of China’s cultural resource curse. Third, the causes and the transmission mechanisms of the cultural resource curse were explained by comparing the influence effects of cultural resources on the development of the cultural industries by regions in terms of four dimensions of influencing factors namely cultural resources, cultural industries input factors, cultural institutions, and economic development environment. In addition, we discuss the crowding-out effect of cultural resource endowment on government fiscal expenditure from educational resources, innovation resources, and public cultural resources.

This study found that the curse of cultural resources exists in most of western China and a few central regions. The impact of cultural resources on the cultural industries is not significant nationwide, but significantly negative in the western region. From the sight of cross-cultural psychology, environmental concern has a weaker association with pro-environmental behavior in some societies (Tam and Chan, 2017), and place attachment makes the development path of cultural industries with certain cultural resource endowment dependence (Zou et al., 2022), cultural and economic disparities between regions appear to be on the rise, with the increasing demand for advantages of modern cultural industries by the decline of more peripheral regions (van der Star and Hochstenbach, 2022). The resource-dependent development model of the cultural industries in the western region attracts more primary labor and squeezes government education spending. It hinders the upgrading of human resources, inhibiting the development of modern innovation in the cultural industries, which is an important reason for the curse of cultural resources in the development of the cultural industries in western China. The research on the curse of cultural resources enriches the theoretical mechanism of cultural industry development. Our research results show that the resource-dependent cultural industries development mode has a crowding-out effect on the upgrading of human resources, which is not conducive to the development of modern cultural industries.

The rest of this study is organized as follows. Section “2. Theoretical background” expounds on the theoretical background, and Section “3. Distribution of the cultural resource curse” introduces the actual situation of the curse distribution of cultural resources in various regions of China. Section “4. Methodology” empirically examines the causes and transmission mechanisms of the cultural resource curse, and finally, Section “5. Conclusion and discussion” provides discusses conclusion and implications.

## 2. Theoretical background

The resource curse theory originated from international trade and later has been widely used in the field of natural resources and energy industries. In recent years, research has developed the curse of financial resources, the curse of political resources, and the curse of tourism resources, breaking the boundaries that the curse of resources originally only applies to natural resources. The relationship between cultural resources and cultural industries has been widely studied by scholars, but there are still some disagreements. For example, existing study reports that rich cultural resources can promote the development of cultural industries (Li and Katsumata, 2020), but some scholars have pointed out that areas rich in cultural resources have not benefit cultural industries with comparative advantages. The lack of consensus hinders the general relationship between cultural resources and the development of cultural industries in the context of regional differences, thereby making it difficult to conclude whether there is a cultural resource curse exists in the cultural industries.

### 2.1. Concept of resource curse

Research on the resource curse began before the 1980s when Singer attempted to explain poverty in resource-exporting countries in terms of deteriorating international terms of trade. Singer (1975) suggests that excessive regional exports of primary commodities have exacerbated the deterioration of supply and demand in the international market. It was not until the 1980s that new growth theories challenged Solow’s convergence process and catch-up hypothesis, and their empirical studies focused on differences in growth rates across countries, finding that regions with abundant natural resources grew more slowly than regions with scarce natural resources (Leite and Weidmann, 1999; Gylfason, 2001). Auty (1994) reports that the economic growth of mineral-rich countries is constrained by mineral resources and who introduces the concept of the resource curse. Later, Stijns (2005) conducts an empirical test on the issue of natural resource abundance and economic growth, proving that natural resource abundance is negatively correlated with economic growth. The resource curse hypothesis exists in some countries that are rich in natural resources.

In addition, research has revealed the causes of the resource curse. For example, Ross (1999) reviews the four most prominent economic explanations for the resource curse, such as declining terms of trade in primary commodities, instability in international commodity markets, and poor economic linkages between resource and non-resource sectors. A disease often referred to as the Dutch disease, evidence of the resource curse comes from the new international economic order, East Asia succeeding with rapid growth in manufacturing exports, and Africa collapsing with declining primary commodities. Likewise, Mehlum et al. (2006) raised concerns over differences in growth performance among asset, resource-rich countries mainly due to how resource rents are allocated through institutional arrangements, they further argued that under producer-friendly institutions, abundant resources attract entrepreneurs into production, which means high growth, benefiting from the specialization of unproductive influence activities under predator-friendly institutions. It can be seen that the causes of the resource curse are closely related to economic development patterns in different periods and institutional differences in different regions.

Much research in the literature of resource curse has emerged not only in natural resources but also in other non-natural resource fields, such as politics and finance. Empirical evidence from Brazil suggests that greater diversion of political resources increases observed corruption and reduces the average education level of mayoral candidates (Brollo et al., 2013). Assessing the impact of the resource curse on bank efficiency from 12 oil-producing countries, the results confirm the existence of the resource curse, and countries that are overly dependent on natural resources tend to have lower financial development (Umar et al., 2021). Few studies have focused on cultural aspects, especially the “cultural resource curse.” The closest study considers the tourism resource curse, where cultural tourism resources are seen as part of cultural resources (Luqman et al., 2022; Wu and Lin, 2022). For example, the study by Khalik et al. (2019) shows that there is a large temporal and spatial variation in the curse of tourism resources in China, and the spatial distribution pattern of the curse of tourism resources is high in the west and low in the east, caused by the spatial heterogeneity of talent scale, transportation accessibility, urbanization, service level, and economic development level.

### 2.2. The concept of cultural resource

The cultural resource is a complex system, and the interpretation of cultural resources usually comes from three perspectives. From the perspective of cultural anthropology, cultural resources are the fruits of civilization or spiritual elements created by human development activities, and methods from applied cognitive anthropology are useful for uncovering cultural consensus and more marginalized perspectives (Brown et al., 2022). From the perspective of cultural labor production, cultural resources are various types of material or spiritual resources that people use to engage in cultural production or cultural activities, labor relations in the context of cultural factors have been studied extensively (Brookes et al., 2011; Luqman et al., 2020), the labor of professional practice can be understood across several dimensions and systems of cultural value (McKay, 2014). From the perspective of cultural economy, cultural resources are factors of production that can be used to develop and create wealth and are various types of resources for cultural industries development activities, Bourdieu’s theory of cultural production fields indicated cultural resources can be used in economic transactions and act as a form of cultural capital (Bourdieu, 1983). King (2011) offers a broad view of cultural resource management that includes archeological sites, cultural landscapes, historic structures, shipwrecks, scientific and technological sites, and objects, as well as intangible resources such as language, religion, and cultural values. From the type of form, cultural resources include tangible and intangible cultural resources or material and spiritual cultural resources; from the type of diachronic, cultural resources include historical and modern; from the type of territory, cultural resources include national and international; from the type of faith, cultural resources include religious and non-religious. The broad diversity and complexity of culture have determined the diversity and complexity of cultural resources. In a conclusion, cultural resources are the material achievements and spiritual wealth created in human cultural activities and are a series of resource systems with cultural attributes that can realize value multiplication. In this study, we tried to quantify the different types of cultural resources in a unified way using an index.

If cultural resources are factors of production and the objective is value production, the value realization of cultural resources is reflected in economic, social, and humanistic dimensions, among others. Xiang divides the evaluation of cultural resource development benefits into humanistic values, such as peculiarity, inheritance, identity, artistic, historical, and social values, and economic values, such as scale, investment, driving forces, industrial base, supporting services and prospect value (Xiang, 2015). Economic growth is a complex evolutionary process that is tightly integrated with sociocultural and political processes, economic and social systems co-evolve through the origination, adoption, and retention of new ideas, and in which creative industries are a key part of this process (Potts, 2009; Zhang et al., 2022). In the dual system and mechanism of China’s cultural industries and cultural undertakings, a large number of public cultural resources are both economic resources and public goods, including cultural heritage and tourism resources, which are not only the supplies for the development of economic industries but also the supplies of social public culture. Services have the dual value of economic and social benefits. In the dual system and mechanism of China’s cultural industries and cultural undertakings, a large number of public cultural resources are both economic resources and public goods, including cultural heritage and tourism resources, which are not only the supplies of economic and industrial development but also the supplies of social public culture. Services have the dual value of economic and social benefits (Saleem et al., 2021). The realization of the value of cultural resources has multiple dimensions, it involves culture, economy, social and wellbeing (Jian et al., 2019), which to a certain extent weakens the value of cultural resources only in economic production.

### 2.3. Cultural resources and cultural industries

Scholars have conducted empirical studies on the input-output efficiency of the cultural industries that showed great variation among different provinces. The overall efficiency of the cultural and creative industries is recognized low in China, environmental factors had significant effects on the development of the cultural industries in each region (Nusrat et al., 2021; Li et al., 2022). There are significant differences in the efficiency of cultural industries inputs and outputs in eastern, central, and western China. Zeng et al. (2016) consider that the scale of companies in the cultural industries is a key factor restricting development, the external environment has a great influence on this efficiency, the efficiency gap between the eastern, central, and western areas is obvious and reflects the degree of the environmental impact on the cultural industries in these regions. Unfortunately, these studies did not consider the impact of the core element of cultural resources on the input-output efficiency of the cultural industries.

A bunch of studies have shown that cultural resources with economic value can significantly contribute to economic growth and cultural industries development, and the results are focused on positive effects. First, all cultural factors in business that can be transformed into economic resources, such as capital and labor can be considered cultural capital (Bourdieu, 1980). Second, the stock of cultural capital can contribute to economic growth, and in the context of sustainable economic growth, cultural capital, human capital, and technical efficiency will become the main driving forces of China’s economic development (Liu and Li, 2019; Bodhi et al., 2022). Third, cultural resources have spatially heterogeneous effects on the development of the cultural industries, cultural resources in tourist destinations are linked to the demand and availability in the medium term (Herrero-Prieto and Gomez-Vega, 2017; Gong et al., 2020).

However, specific cultural resources or cultural capital do not always show a positive association with the cultural industries. Different pathways of influence, heterogeneity of cultural resources, different market environments, and changing industrial factor conditions may result in differentiated development outcomes in the certain cultural industries. In classical economic growth theory and modern economic growth theory, cultural capital can indirectly act on economic development by constraining technological choice and institutional choice (David, 1975; Chang, 2011; Bisin et al., 2021; Qi et al., 2021). Meanwhile, more attentions are paid to the links between creative economy and local development, inequalities of opportunity and businesses of spiritual cultural capital have different influences on economic development from the perspective of cultural capital heterogeneity (Tubadji, 2014; Ballet et al., 2015), development of a creative economy can form an integral part of any attempt to redress inequality, provided that the process also brings about broader structural changes to ensure that creative workers are themselves not disadvantaged in relation to other workers (Boccella and Salerno, 2016), yet, the economy and the cultural industries of western China are still underdeveloped, the explanations for this are mainly due to the small scale of the enterprise, insufficient conditions to create demand, shortages of talent, and lack of investment capital for the cultural industries development (Fan and Xue, 2018; Fan and Zhang, 2019).

## 3. Distribution of the cultural resource curse

In the theory of the natural resource curse, the mainstream view describes the negative effect of natural resources on economic growth. Before proving the utility of the positive or negative effects of cultural resources on cultural industries development, we construct the cultural resource curse coefficient by referring to the natural resource curse coefficient (Lu et al., 2019; Yang and Song, 2019). The curse coefficient of cultural resources refers to the ratio of the proportion of regional cultural resources to the total national cultural resources to the proportion of the added value of the cultural industries in the region to the added value of the cultural industries in the country. This resource curse coefficient essentially explains the degree of comparative advantage of regional cultural resources relative to the development of cultural industries, it is used to measure the degree of deviation between industrial development and resource endowment in the region. The cultural resource curse coefficient is calculated using the following equation:


(1) $$C\,R\,C_{i}=\frac{C\,R_{i}/\sum_{i=1}^{n}C\,R_{i}}{C\,u\,l_{i}/\sum_{i=1}^{n}C\,u\,l_{i}}$$

The cultural resources curse _*CRC_i_*_ indicates the deviation of cultural resources from the development of cultural industries in the region, _*n*_ indicates the number of provincial administrative units in China, Cultural resources endowment index _*CR_i_*_ indicates the cultural resources endowment of the region *_*i*_*, _*Cul_i_*_ indicates the added value of the cultural industries in the region _*i*_.

When the cultural resource curse coefficient is larger than 1, the proportion of the region’s cultural resource endowment in the country is larger than the proportion of the region’s cultural industries added value in the country, the region’s cultural resource endowment advantage is greater than the region’s cultural industries development advantage, and the cultural resource endowment advantage shows a negative deviation from the cultural industries development advantage, which means that the region has a cultural resource curse. The larger the cultural resource curse coefficient is, the more advantage a region’s cultural resources have over its cultural industries, the higher the deviation of the development advantage from the endowment advantage, and the more serious the cultural resource curse is. When the cultural resource curse coefficient is less than 1, the opposite is true.

### 3.1. Cultural resource endowment index

Drawing on the indicators of cultural resources (King, 2011; Xiang, 2015; Zhou and Liu, 2019), we construct the cultural resource endowment index *CR*_*i*_ with nine secondary indicators in five dimensions, including public cultural resources, intangible cultural heritage resources, tourism resources, local language resources, and famous villages and towns resources. The composition of the cultural resource endowment index is shown in Table 1. Data are drawn from the China Statistical Yearbook of Culture and Related Industries, China Statistical Yearbook of Culture and Tourism, and the official websites of the Ministry of Housing and Urban-Rural Development of the People’s Republic of China, China Intangible Cultural Heritage Website, and China Tourism Website. The cultural resource endowment index of each province in China is calculated by using the weighted TOPSIS method (Jahanshahloo et al., 2006).

**TABLE 1:** Index system of cultural resource endowment.

Primary indicator	Secondary indicator
Public cultural resources	Number of libraries
	Number of museums
	Number of books in library
	Number of cultural centers
Intangible cultural heritage resources	Number of national-level intangible cultural heritage items
	Number of provincial-level intangible cultural heritage items
Tourism resources	Number of scenic spots of grade A or above
Local language resources	Number of dialects
Famous villages and towns resources	Number of famous villages and towns of the ministry of housing and construction

### 3.2. Measurement of the cultural resource curse coefficient

According to Eq. (1), based on the data of the cultural resource endowment index and added value of the cultural industries, we measured the results of the curse coefficient of 29 provincial administrative units in China for 20 years from 2000 to 2019 (due to missing data for Xinjiang and Tibet, these two provinces are not included in the calculation) and calculated to get the average of 20 years for each province. To facilitate the comparison of the results, the country was divided into four subdivisions (Lu et al., 2019), including no-resource curse zone, resource curse edge zone, resource curse high-risk zone, and resource curse severe zone. The regional distribution of the curse is shown in Table 2.

**TABLE 2:** Regional distribution of the cultural resource curse in China.

Resource curse distribution	Threshold	Provincial area	Characteristic description
No resource curse zone	*CRC*_*i*_ < 1	Shanghai, Beijing, Guangdong, Tianjin, Zhejiang, Jiangsu, Chongqing, Fujian.	The development advantage of the cultural industries is stronger than that of the local cultural resource endowment, and there is no resource curse.
Resource curse edge zone	1≤*CRC*_*i*_ < 2	Hunan, Shandong, Hainan, Sichuan, Jilin, Hubei, Anhui, Liaoning, Ningxia, Jiangxi, Shaanxi, Guangxi, Hebei.	The advantages of cultural resource endowments are slightly stronger than the advantages of cultural industries development, but the degree of deviation between cultural industries development and cultural resource endowment is not high.
Resource curse high-risk zone	2*CRC*_*i*_ < 4	Henan, Yunnan, Heilongjiang, Inner Mongolia, Guizhou, and Shanxi.	There is a more serious deviation between cultural industries development and cultural resource endowment. The advantages of cultural resources have not been transformed into an advantage in the cultural industries.
Severe resource curse zone	4≤*CRC*_*i*_	Gansu, Qinghai.	Cultural industries development is very weak and seriously deviates from the local cultural resource endowment conditions.

In the no-resource curse zones, the cultural resource curse coefficient is less than 1, and the advantages of regional cultural industries development are greater than those of local cultural resource endowments. There are 8 provinces (cities), such as Shanghai, Beijing, Guangdong, and Fujian, in the no-resource curse zone. The no resource curse zone is mainly distributed in municipalities directly managed by the central government and some eastern regions, which have a high level of economic development, strong industrial foundation, superior market environment, and strong cultural industries development. Cultural industry in these regions is driven by market-oriented conditions, factor conditions, and innovation levels rather than cultural resources.

In the resource curse edge zone, the cultural resource curse coefficient is between 1 and 2, the advantages of cultural resource endowments in the region are slightly stronger than the advantages of cultural industries development, but the two do not show serious divergence. There are 13 provinces (autonomous regions), such as Hunan, Shandong, and Hainan, in the resource curse edge zone, accounting for approximately half of provincial units in China. Resource curse edge zones, which do not show obvious regional distribution characteristics, are distributed in the eastern, central, and western regions, where the development of cultural industries and cultural resource endowment is relatively balanced.

Regions with high risk and severe cultural resource curses have coefficients greater than 2, the development of cultural industries within these regions seriously deviates from the local cultural resource endowments, and the advantage of cultural resources is greater than the advantage of cultural industries development. The provinces with high-risk resource curses are mainly distributed in the western region and the two central regions of Henan and Shanxi, among which six provinces (autonomous regions), including Henan, Yunnan, and Heilongjiang, have resource curse coefficients between 2 and 4. Generally, their cultural resource endowments have not been transformed into the advantage of cultural industries development. Two provinces, Gansu and Qinghai, have resource curse coefficients greater than 4, at the most severe level. The development of their cultural industries is severely lagging, and the development of cultural industries and cultural resource endowment conditions are seriously inverted.

The regional distribution shows that the cultural resource curse generally exists in economically underdeveloped regions, while it generally does not exist in economically developed regions. This phenomenon suggests that the development of cultural industries in a region has a strong correlation with the local level of economic development, while it is only weakly correlated with the local cultural resource endowment.

## 4. Methodology

Many categories of industrial economy are in the industrial ecology that has spatial heterogeneity. Accurate identification of different regional industrial ecology, and reasonable allocation of resources are conducive to the sustainable industrial development, while unreasonable allocation of resources hinders industrial development (Xu et al., 2022). To further investigate the intrinsic causes of the cultural resource curse, we place cultural resources into the economic ecology of cultural industries, and we first empirically examine the influencing factors of cultural industries and then conduct an empirical test on the transmission mechanism of the cultural resource curse in western China, where the cultural resource curse is severe.

### 4.1. Research design and model setting

Based on extant study in the literature of resource curse (Atkinson and Hamilton, 2003; Fleming et al., 2015; Perez-Sebastian and Raveh, 2016; Corrocher et al., 2020; Hu et al., 2020), this study constructs an empirical model of the influence factors of the cultural industries, which covering cultural resources, and empirically examines the influence effect of cultural resources on the cultural industries by region. In this model, the added value of the cultural industries is taken as the explained variable, cultural resources as the core explanatory variable, and control variables covering cultural industries input factors, cultural institutional mechanism factors, and economic environmental factors. For the sake of data smoothness, we performed logarithmic treatment on some variables. The empirical model is as follows:


$$l\,n\,\_\,C\,u\,l_{i,t}=\beta_{0}+\beta_{1}\,C\,R_{i,t}+\beta_{2}\,I\,n\,p\,u\,t_{i,t}+\beta_{3}\,I\,n\,s\,t\,i\,t\,u\,t\,i\,o\,n_{i,t}$$

(2) $$+\beta_{4}\,E\,c\,o\,n\,o\,m\,i\,c_{i,t}+\varepsilon_{i,t}$$

In the equation, the subscript *i* denotes the provincial administrative unit, the subscript _*t*_ denotes the year, _*Cul_i,t_*_ denotes the added value of the cultural industries in the year _*t*_ in the region _*i*_, _*CR_i,t_*_ denotes the cultural resources in year *t* in the region _*i*_, _*Input_i,t_*_, _*Institution_i,t_*_, and _*Economic_i,t_*_ respectively, denotes the control variables of the three dimensions of cultural industries input, cultural institutional mechanism, and economic environment, _*β*_0__ denotes the constant term, _*β*_1__, _*β*_2__, _*β*_3__, and _*β*_4__ denotes the measured coefficients, and _*ε*_i,t__ denotes the random disturbance term.

The model applied panel data from 29 provincial administrative units in China over 20 years from 2000 to 2019, and panel data regressions usually involve mixed POOL models, fixed-effects FE models, and random-effects RE models. At the prior tests, we applied relevant methods such as F tests, BP Lagrange multiplier tests, and Hausman tests can determine the appropriate model settings, and to overcome heteroskedasticity and autocorrelation between variables, we include clustering robustness standard errors Robust in the model. The description of variables is presented in Table 3, the data are obtained from the China Culture and Related Industries Statistical Yearbook, China Statistical Yearbook, Marketization Index, and CNKI Data Service Platform.

**TABLE 3:** Description of variables.

Variable type	Variable name	Variable symbol	Variable meaning
*Explained variable*	Industry value added	In Cul	Logarithm of value added of cultural industries
*Explanatory variable*	Cultural resource	In CR	Logarithm of cultural resource endowment index
*Control variable*	Labor	In CL	Logarithm of labor input in cultural industries
	Financial investment	In CF	Logarithm of financial investment in cultural industries
	Cultural institution	PC	Share of cultural business expenses in fiscal expenditures
	Cultural legal person	In LP	Logarithm of the number of legal persons in culture, sports and entertainment industry
	Economic development level	In PGDP	Logarithm of GDP per capita
	Marketization	MI	FanGang marketization index
	Degree of openness	OP	Number of accesses to foreign products

Cultural resources can influence the development of the cultural industries by forming cultural commodities available for transformation into cultural capital and forming a cultural atmosphere. Cultural resource variables are based on the calculation of the cultural resource endowment index in the previous section. There are two input factors of the cultural industries, including labor input and financial investment, and both can affect the cultural industries directly. Chinese cultural institutional mechanism has the dual attributes of economic and social benefits, which are related to the development of the cultural industries. In this study, we include two variables: (1) the proportion of cultural undertakings expenditure to fiscal expenditure and the number of legal persons in the cultural, sports and entertainment industry; (2) the proportion of cultural expenditure to fiscal expenditure can better reflect the structure of the cultural institutional mechanism, the number of legal persons in the cultural, sports and entertainment industry reflects the scale of regional cultural entities. The level of local economic development affects the development of the cultural industries through the indirect effects of cultural demand, cultural consumption, and cultural production. We take per capita GDP to measure, the higher the level of local economic development, the stronger the demand and preference for culture and the more the economy can promote the development of the cultural industries. Considering the influence of the market environment on the development of the cultural industries, we followed the measurement of Fan et al. (2011) to measure the degree of market development. Additionally, the degree of openness is constructed by the number of foreign investment entities with access to products in each industry. Summary statistics are presented in Table 4.

**TABLE 4:** Summary statistics.

VarName	Obs.	Mean	*SD*	Min.	Max.
In Cul	580	2.39	0.600	0.89	3.76
In CR	580	−1.20	0.366	−3.00	−0.12
In CL	580	1.51	0.735	−1.00	3.15
In CF	580	2.01	0.688	0.00	3.38
In LP	580	3.52	0.514	1.48	4.80
PC	580	0.46	0.118	0.23	0.90
In PGDP	580	4.33	0.371	3.44	5.21
MI	580	6.35	1.950	2.33	11.71
OP	580	4.61	5.407	0.44	24.46

### 4.2. Empirical analysis and results

#### 4.2.1. Stepwise regression

Table 5, we test the relationship between cultural resources and cultural industries, the stepwise regression results of Model 1 to Model 4. The result in Model 1 shows a significant positive correlation between cultural resources and cultural industries, but this is not sufficient to explain the influence of cultural resources on the development of cultural industries under different conditional environments. The results of the stepwise regression of Models 2, Model 3, and Model 4 show that cultural resource endowment does not have a significant impact on cultural industries development and that the results are robust. The results verify the standpoint that the association between regional cultural industries development and cultural resource endowment is weak in the analysis of the resource curse coefficient. Moreover, Model 3 and Model 4 indicate that cultural institutions have a significant inhibitory effect on cultural industries development, constraining the economic value of the cultural industries. Similarly, it happens in Italy, where heritage and intangible capital are important driver for economic and socio-cultural development, but the absence of systemic and integrated vision of cultural sector brings economic and social benefits which are inadequate to the fields potentiality (Simeon and Martone, 2014).

**TABLE 5:** Results of stepwise regression.

	(1)	(2)	(3)	(4)
	In Cul	In Cul	In Cul	In Cul
In CR	1.306**	1.180	0.101	0.243
	(5.684)	(0.869)	(0.737)	(1.784)
In CL		0.342*	0.201	0.104
		(2.048)	(1.441)	(0.704)
In CF		0.484**	0.354**	0.229*
		(2.914)	(3.022)	(2.381)
In LP			0.359**	0.175*
			(3.390)	(2.148)
PC			−0.670**	−0.751**
			(−3.685)	(−4.299)
In PGDP				0.451**
				(3.701)
MI				0.043**
				(2.883)
OP				0.003
				(0.347)
Cons	3.953**	1.115**	0.546**	−0.441
	(15.150)	(4.661)	(3.006)	(−1.149)
Model setting	RE	RE	RE	RE
R^2^	0.231	0.771	0.789	0.834
N	580	580	580	580

*T*-values in parentheses, *p < 0.05, **p < 0.01.

The result of Model 4 indicates that the level of regional economic development and the degree of marketization, financial investment, and the number of legal persons engaged in the culture industry can significantly promote its development. Therefore, in the more economically developed eastern regions, the economic environment, the level of marketization, and the factor conditions for the development of the cultural industries are more adequate, the transformation and utilization of cultural resources are higher, and the curse of cultural resources does not occur. Meanwhile, in our model, the effects of cultural labor input and market openness on cultural industries development are not significant for the whole country. One possible reason is that China’s cultural industries is not at the stage of talent-driven and open development, the utility level of cultural labor input and the degree of openness of the market are still low and do not have a significant effect on the development of the cultural industries.

#### 4.2.2. Subregional regression

Nowadays, the development and utilization of cultural resources should satisfy cultural and economic needs to assess the key conditions in the context of place attachment, the results of subregional regressions for the east, central and west regions are presented in Table 6, we can see the large differences in influence factors for cultural industries in different regions. Cultural resources have a significant positive impact in the east and a significant negative impact in the western regions. Due to the regional heterogeneity in the eastern, central, and western regions, the different regional economic development levels, market conditions, factor conditions, innovation, and creativity capabilities cause different levels of development and utilization of cultural resources. The strong development and utilization of cultural resources in the east show a significant positive impact of cultural resources on cultural industries and the weak development and utilization of cultural resources in the western regions do not. This finding supports the regional distribution results of the regional cultural resource curse.

**TABLE 6:** Results of sub-regional regression.

	(4) National region	(5) Eastern region	(6) Central region	(7) Western region
	In Cul	In Cul	In Cul	In Cul
In CR	0.243	0.424*	−0.173	−0.081*
	(1.784)	(2.441)	(−2.005)	(−2.092)
In CL	0.104	−0.021	0.388**	0.158*
	(0.704)	(−0.157)	(5.156)	(2.175)
In CF	0.229*	0.278	0.005	0.201
	(2.381)	(1.649)	(0.045)	(1.916)
In LP	0.175*	0.247*	−0.250**	−0.082
	(2.148)	(1.986)	(−3.905)	(−1.150)
PC	−0.751**	−0.829**	−0.570*	0.048
	(−4.299)	(−2.987)	(−2.061)	(0.210)
In PGDP	0.451**	0.657**	0.857**	0.652**
	(3.701)	(2.661)	(8.704)	(4.075)
MI	0.043**	0.015	0.034	0.061**
	(2.883)	(0.773)	(1.826)	(3.062)
OP	0.003	0.012	0.092**	−0.014
	(0.347)	(1.338)	(2.638)	(−1.773)
Cons.	−0.441	−1.165	−1.221**	−1.248*
	(−1.149)	(−1.898)	(−3.277)	(−2.477)
Model setting	RE	RE	FE	FE
R^2^	0.834	0.781	0.745	0.650
N	580	220	160	200

*T*-values in parentheses, *p < 0.05, **p < 0.01.

In addition, there are different influences on the drivers of cultural industries in the eastern, central, and western regions. In the eastern region, the level of local economic development (GDP per capita) and the number of cultural legal persons have a significant positive influence on the development of the cultural industries, the cultural institution system has a significant negative influence on the development of the cultural industries, the cultural industries labor input, financial investment, the degree of marketization and openness show non-significant influences on the development of the cultural industries.

In the central region, the level of local economic development (such as GDP per capita), the cultural industries’ labor input, and the degree of openness has significant positive effects on the development of the cultural industries, while cultural legal persons, the cultural institution system have significant negative effects on the development of the cultural industries. Cultural industries’ financial investment and the degree of marketization have no significant effects on the development of the cultural industries.

In the western region, the GDP per capita of the local economic development level, the degree of marketization, and the labor input in cultural industries have significant positive effects on the development of the cultural industries, while cultural resources have significant negative effects on the development of the cultural industries. The financial investment in the cultural industries, the number of cultural legal persons, the cultural institution system, and the degree of openness in the development of the cultural industries are not significant.

### 4.3. Transmission mechanism of cultural resource curse

The theory of the natural resource curse hypothesis suggests that regions with the resource curse are often accompanied by a crowding-out effect on other resources such as human resources, technology, and innovation resources and resources in the manufacturing sector (Deng et al., 2014; Wen and Jia, 2022). In the case of the cultural industries, we explore how the cultural resource curse is transmitted in the western region from the crowding-out effect of cultural resources on human resources, innovation resources, and cultural utility resources.

Cultural resources have cultural attributes, regional culture has an effect on regional population and cultural labor supply on the one hand and human resources through cultural concepts and education on the other hand (Vinogradov and Kolvereid, 2007; Yong, 2019). Meanwhile, human resources can significantly influence the growth of cultural industries (Sonn et al., 2019). Therefore, we take local cultural industries’ labor and fiscal education expenditure as two indicators to measure human resources. To eliminate the scale differences between regions, we thus constructed two measurements, the number of local cultural industries employees as a share of the resident population (E_CL_) and the share of local education expenditure as a share of fiscal expenditure (Edu).

Cultural resources also constitute the basis for the operation of creative industries and obtain their value from culture (Deng, 2011; Mao, 2020; Wu and Lin, 2021). The level of creativity is one of the key factors in promoting the cultural industries (Xu et al., 2021). We choose local R&D expenditure in science and technology as a measure of local creativity, the default assumption is that the higher the expenditure on scientific research in a region, the stronger the innovation capacity and thus the higher the creativity. Additionally, for the sake of the relative smoothness of interregional comparison, the share of local expenditure on scientific research to fiscal expenditure (E_R&D_) is applied as the measurement variable.

As mentioned in the previous theory, on the one hand, cultural resources have dual attributes of cultural industries and cultural undertakings. Cultural resources are among the components of cultural undertakings. On the other hand, the above theoretical and empirical discussion shows that cultural undertakings generally have inhibitory effects on the development of the cultural industries. We choose the share of funding for local cultural undertakings in fiscal expenditure (PC) as the measurement variable.

To test the robustness of the main empirical results, we used the degree of marketization (MI) and the degree of openness (OP) as control variables in the model and obtained empirical results demonstrating how the cultural resources curse is transmitted in the western region of China, as shown in Table 7.

**TABLE 7:** Results of the transmission mechanism.

	E_CL_	Edu	E_R&D_	PC
In CR	0.179**	−0.051*	−0.005	−0.054
	(3.860)	(−1.977)	(−0.612)	(−1.840)
Control variable cons.	YES 0.223** (5.516)	YES 0.207** (4.093)	YES 0.039 (1.869)	YES 0.380** (6.758)
Model setting	RE	RE	RE	RE
R^2^	0.579	0.316	0.040	0.028
N	200	200	200	200

*T*-values in parentheses, *p < 0.05, **p < 0.01.

The empirical results of the transmission mechanism indicate the following: cultural resources and cultural industries labor are positively related. Cultural resources can encourage the agglomeration of cultural workers and significantly promote the development of cultural industries by promoting the growth of cultural industries workers. Cultural resources are significantly negatively correlated with inputs from education resources. In the typical case of Sweden, urban, natural and cultural qualities are different sources of regional attractiveness and influence the growth of human capital (Backman and Nilsson, 2018), but the Chinese western regions are peripheral layer of the cluster innovation network in China (Liu et al., 2022), as a result, the western regions are not attractive to skilled and well-educated people. The Chinese western regions with abundant cultural resource endowments are often enable policymakers to rely on cultural resources for culture-related industries development and income growth. In the context of Chinese western regions, the important value of education, as well as human resources for cultural industries development are neglected, which in turn hinders the transformation, upgrading, and innovative development of the cultural industries.

In reality, the cultural industries in western regions tends to attract more low-skilled and low-educated laborers, who often engage in resource-intensive and labor-intensive primary cultural industries too early or too long, reducing their own needs for educational development and inhibiting local education expenditures and human resource upgrading. Moreover, in regions with cultural resource endowment advantages, the government tends to pour more finances into the utilization, development, and protection of cultural resources, forming a certain crowding out of expenditures for education, thus hindering the upgrading of local human resources. Cultural resources do not have a significant correlation with either the level of creativity or cultural undertakings. In contrast to the natural resource curse, the cultural resource curse appears to be different to the way natural resources crowd out science and technology innovation and manufacturing, while cultural resources may not crowd out cultural creativity or cultural undertakings.

The transmission mechanism of the cultural resource curse in western China suggests that an important reason for the cultural resource curse in the western region is that the cultural resource endowments attract more primary labor, while there is a local crowding-out effect on educational resources, which inhibits the upgrading of local human resources and thus hinders the development of the cultural industries.

## 5. Conclusion and discussion

The study attempts to demonstrate the existence of the cultural resource curse. Draw on the resource curse theory, we measured the cultural resource curse based on 29 provinces in China from 2000 to 2019. To further explore the causes and possible transmission mechanism of the curse of cultural resources, we examine the influence of cultural resources on cultural industries and the crowding-out effect of cultural resources on other resources. The findings show that the resource curse is more serious in the economically less developed western region, and cultural resources harm the cultural industries in the western region. Refereeing from the experience of the curse of Chinese cultural resources under the modern development of the cultural industries, the key factors that determine the development of the cultural industries include economic factors, industrial input factors, and market environment factors, rather than depending on the degree of abundance of cultural resource endowment. We further found that the curse of western cultural resources is because western cultural resources attract a large primary labor force, which has a crowding effect on educational resources and hinders the upgrading and innovation of cultural industries.

### 5.1. Theoretical implications

Our research has certain theoretical significance. The study on the curse of cultural resources expands a new theoretical framework for the research on the relationship between cultural resources and the development of cultural industries. This study makes three theoretical contributions. First of all, cultural resources are a complex system of public cultural resources, heritage resources, tourism resources, language resources, etc., we define the scope of cultural resources from a broad perspective, which provides a research perspective for the analysis of the components of cultural resources and lays the foundation for the study of the curse of cultural resources. Second, our study confirmed the existence of the curse of cultural resources in the development of cultural industries, the theory of place attachment and cultural field illustrate the regional differences when cultural industries are taken account into an industrial ecosystem, so cultural resources have different influence effects and transmission mechanisms on the regional development of cultural industries. Specifically, we compared the impact of cultural resources on the development of cultural industries in China from four dimensions: cultural resources, industrial factor input, cultural institutional factors, and economic development level factors. We found that the impact of cultural resources is unfavorable to the development of western cultural industries. The empirical results are helpful to the resource-dependent development model of cultural industries, which is not conducive to the formation of comparative advantages of cultural industries development. Third, Examining the crowding-out effect of cultural resources on other resources from the aspects of government spending on educational resources, innovation resources, cultural undertakings resources, etc., provides a research direction for exploring the causes of the cultural curse resources.

### 5.2. Practical implications

The research results have important practical significance for clarifying the relationship between cultural resources and cultural industries development. The study results have a reasonable strategic focus on the development of the cultural industries, balance the economic and social benefits of cultural resources, and improve the utilization efficiency of cultural resources, the development and utilization of cultural resources should not only balance the relationship between culture and economy, but the development of cultural industries should also pay attention to cross-regional and cross-cultural territorial diversity.

First, it is often difficult to achieve ideal results in the development of cultural industries by developing cultural resources in economically underdeveloped areas. Blindly developing existing cultural resources or vigorously developing new cultural resources such as scenic spots and cultural facilities cannot effectively promote the development of local cultural industries. Excessive development and utilization of cultural resources result in a waste of resources, crowding out an investment that can be used for educational resources, which in turn inhibits the upgrading of human resources, thereby hindering the development of cultural industries.

Second, the strategic focus of cultural industries development in different regions is different. All regions should give top priority to economic development, promote the increase of residents’ income, and take the expansion of cultural consumption demand as the priority for the development of cultural industries. In addition, we suggest that the eastern region should take the development of cultural resources and the expansion of cultural legal persons as the strategic focus of cultural industries development. The central region should promote the opening of the market and increase the input of the labor force in the cultural industries as a strategy for the development of the cultural industries. The western region should focus on improving the degree of marketization and promoting the growth of human resources in the cultural industries.

Third, given the dual system and mechanism of the country’s cultural industries and cultural undertakings, in the economically developed regions, such as the eastern region, the degree of marketization is high, and it is easier to stimulate the economic benefits of cultural resources. The policy of accelerating the market-oriented reform of cultural undertakings is reasonable, but in economically underdeveloped regions such as the west, the vitality of the cultural economy and the guarantee of public cultural services still mainly depend on cultural undertakings. Before the cultural market has matured, it is not appropriate to carry out the market-oriented reform of cultural undertakings prematurely.

### 5.3. Limitations and future directions

Despite novel contributions, our research is not without limitations. First of all, cultural resources are a broad concept and system. Our definition of cultural resources may be biased toward the endowments left by historical and traditional cultural resources such as geography and humanities. Art and cultural resources such as music and painting, as digital cultural resources such as media and digital collections, are not included, and the sampling scope of future cultural resources is still recommended for future scholars to investigate, at the same time, cross-environmental and cross-cultural psychology perspective is also worth to more concern. In our empirical models, we consider the four dimensions of the cultural industries, which are more suitable for the development characteristics of China’s cultural industries. Future researchers should extend our findings with more cultural dimensions appropriate to the other regions or countries. In addition, control variables can be selected based on the context and characteristics of the region. For example, in the European Union (Moore, 2014) or the United Kingdom (Garnham, 2006), cultural industries are called as creative industries, which mainly highlight economic activities which are concerned with the generation or exploitation of knowledge and information, and thus control variables related to creativity can be selected, and cultural industries in Japan and South Korea tend to focus on the private sector (Otmazgin, 2011; Li X. et al., 2021; Wang et al., 2022; Zhou et al., 2023). Furthermore, technology and copyright play an increasingly important role in cultural industries, and future research should consider these explanatory variables. Our research has confirmed the existence of the curse of cultural resources in the development of cultural industries. However, how to break the curse of cultural resources and transform the curse of cultural resources into the blessing of cultural resources is still the suggestion of future scholars.

## Data availability statement

The original contributions presented in this study are included in the article/supplementary material, further inquiries can be directed to the corresponding author.

## Author contributions

JZ contributed to the design of research themes, research framework and the theory, and to the summary of the conclusion. ZX contributed to collecting data and the empirical study, and to the validation of the conclusion. YL contributed to the literature review and edited the article. All authors discussed the results and commented on the manuscript.
